# Right Lateral Cerebellum Represents Linguistic Predictability

**DOI:** 10.1523/JNEUROSCI.3203-16.2017

**Published:** 2017-06-28

**Authors:** Elise Lesage, Peter C. Hansen, R. Chris Miall

**Affiliations:** ^1^Department of Experimental Psychology, Ghent University, 9000 Ghent, Belgium, and; ^2^School of Psychology, University of Birmingham, Birmingham B15 2TT, United Kingdom

**Keywords:** cerebellum, fMRI, language, nonmotor, phonological working memory, prediction

## Abstract

Mounting evidence indicates that posterolateral portions of the cerebellum (right Crus I/II) contribute to language processing, but the nature of this role remains unclear. Based on a well-supported theory of cerebellar motor function, which ascribes to the cerebellum a role in short-term prediction through internal modeling, we hypothesize that right cerebellar Crus I/II supports prediction of upcoming sentence content. We tested this hypothesis using event-related fMRI in male and female human subjects by manipulating the predictability of written sentences. Our design controlled for motor planning and execution, as well as for linguistic features and working memory load; it also allowed separation of the prediction interval from the presentation of the final sentence item. In addition, three further fMRI tasks captured semantic, phonological, and orthographic processing to shed light on the nature of the information processed. As hypothesized, activity in right posterolateral cerebellum correlated with the predictability of the upcoming target word. This cerebellar region also responded to prediction error during the outcome of the trial. Further, this region was engaged in phonological, but not semantic or orthographic, processing. This is the first imaging study to demonstrate a right cerebellar contribution in language comprehension independently from motor, cognitive, and linguistic confounds. These results complement our work using other methodologies showing cerebellar engagement in linguistic prediction and suggest that internal modeling of phonological representations aids language production and comprehension.

**SIGNIFICANCE STATEMENT** The cerebellum is traditionally seen as a motor structure that allows for smooth movement by predicting upcoming signals. However, the cerebellum is also consistently implicated in nonmotor functions such as language and working memory. Using fMRI, we identify a cerebellar area that is active when words are predicted and when these predictions are violated. This area is active in a separate task that requires phonological processing, but not in tasks that require semantic or visuospatial processing. Our results support the idea of prediction as a unifying cerebellar function in motor and nonmotor domains. We provide new insights by linking the cerebellar role in prediction to its role in verbal working memory, suggesting that these predictions involve phonological processing.

## Introduction

The cerebellar role in language and cognition has become increasingly apparent over recent decades (Strick et al., 2009). Patient and functional imaging data show that cerebellar regions contributing to language and cognition are largely confined to the posterolateral cerebellum (hemispheric portions of Lobule VII, consisting of Crus I and Crus II). These regions are reciprocally connected with supramodal neocortical areas, as demonstrated using tracer studies in nonhuman primates (Kelly and Strick, 2003) and by functional connectivity MRI in humans (Buckner et al., 2011; Bernard et al., 2012). A wealth of neuroimaging studies report right posterolateral cerebellar activation in studies that probe language (Stoodley and Schmahmann, 2010; Price, 2012) and working memory processes (Desmond et al., 1997; Hayter et al., 2007; Stoodley and Schmahmann, 2009; Keren-Happuch et al., 2012). However, the functional contribution of the cerebellum in language remains unclear. In motor control, the cerebellum is thought to acquire and store internal models of the motor system. These internal models predict upcoming reafferent sensory input and these continuous short-term predictions allow for fluent movements and efficient error correction (Miall et al., 1993; Wolpert and Miall, 1996; Miall, 1998). Based on the homogeneous cerebellar cytoarchitecture, some investigators have argued that the cerebellar role in nonmotor functions is like that in motor control, performing similar operations on more abstract inputs (Bloedel, 1992; Ramnani, 2006; Ito, 2008). Therefore, extrapolating from the internal model motor theory of the cerebellum, the posterolateral areas of the cerebellum might support short-term prediction of future linguistic stimuli.

A testable hypothesis can be derived from this proposal: the cerebellum, specifically the right Crus I/II, should be differentially engaged when processing highly predictable versus unpredictable language. Consistent with this notion, online prediction of upcoming sentence content is slowed after perturbation of the right cerebellum with transcranial magnetic stimulation (TMS; Lesage et al., 2012) and modulated by electrical stimulation (tDCS Miall et al., 2016; D'Mello et al., 2017). In addition, fMRI studies have reported right cerebellar recruitment in conditions where linguistic prediction is possible (Desmond et al., 1998; Moberget et al., 2014). However, it has been difficult to manipulate linguistic prediction without also introducing differences in speech production processes, linguistic properties of the stimulus, task difficulty (working memory load), or outcome evaluation (prediction error); each of these processes have been shown to recruit the posterior cerebellum (Petersen et al., 1989; Floyer-Lea and Matthews, 2004; Fedorenko et al., 2010; Stoodley et al., 2012; Grimaldi et al., 2014; Argyropoulos, 2016; Moberget and Ivry, 2016). To date, no fMRI study has been able to capture cerebellar responses to linguistic prediction during comprehension while controlling for these confounds.

Here, we manipulated the predictability of sentences in an event-related fMRI design and tested whether the hemodynamic response in right Crus I/II covaried with predictability. Critically, the time at which a prediction is made was isolated from the outcome of the sentence and from the contextual information that allows a prediction to be made. In addition, we explored whether the cerebellar roles in working memory and linguistic prediction could be reconciled; for example, perhaps linguistic prediction requires short-term storage of semantic, phonological, or orthographic representations. Therefore, we further assessed whether cerebellar regions identified in the predictive task were engaged in three additional fMRI tasks that capture semantic, phonological, and orthographic (visuospatial) working memory.

## Materials and Methods

### 

#### Participants

Eighteen right-handed volunteers (4 male, average age 21 years, range 18–27 years) participated in two fMRI sessions. One male subject was excluded from the second session and from all data analysis due to severe signal dropout in the lateral cerebellum. All participants were native English speakers; none were fluent in any other language. Participants were remunerated for their time. Written informed consent was obtained for each participant. This study was approved by the local ethics committee at the University of Birmingham and was performed in accordance with the guidelines set out in the Declaration of Helsinki (1964).

#### Prediction task

Participants silently read visually presented sentences with varying degrees of predictability and pressed an MR-compatible response button to indicate the plausibility of the sentence. Participants were not informed that the predictability of sentences was relevant and were merely instructed to read the words presented on the screen and judge whether the outcome of the final item was likely given the context. The task consisted of 78 trials, each presenting a unique item (context sentence + stem of a second sentence). Thirty-three items were taken from a study by Fitzsimmons and Drieghe (2013) and altered to better suit this fMRI design; 45 items were newly constructed. A behavioral pilot experiment in an independent sample of 43 participants had determined the items' predictability (cloze probability). Cloze probability can be defined as the probability that a sentence will be completed with a given target word (e.g., a cloze probability of 0.90 indicates that 90% of participants will complete the item with the same target word). Cloze probability was used as a continuous parametric modulator in behavioral and fMRI analysis. We also categorized items as neutral (cloze probabilities between 0 and 0.40; 27 items), semipredictable (cloze probabilities between 0.40 and 0.70; 25 items), and predictable (cloze probabilities between 0.70 and 1.00; 26 items). These discrete levels of predictability were used for easier visualization of the results; all analyses were conducted with cloze probability as a continuous variable.

**Figure 1.**
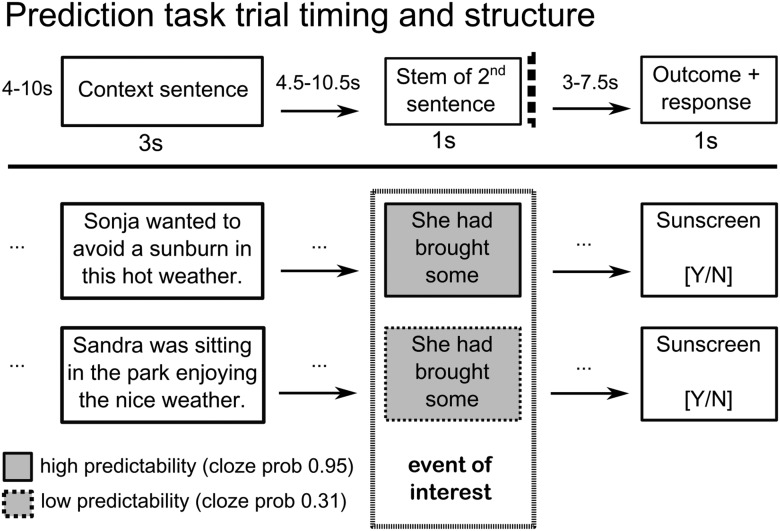
Trial structure of the prediction task. The stem and the outcome stimuli are matched for high- and low-cloze trials and the context sentences are matched for length; here, two items with the same stem in which one has a very predictable sentence ending (cloze probability 0.95) and the other does not (cloze probability 0.31). Three temporal events are modeled independently in the analysis: the context (3 s), the stem (1 s), and the outcome (1 s). Ellipsis indicate variable delay/temporal jitter.

Three temporal events per trial were modeled independently to allow separate estimation of the BOLD response to these events (Fig. 1). The first was the presentation of a context sentence (CONTEXT), which appeared on the screen for 3 s (e.g., “Sonja wanted to avoid a sunburn in this hot weather.”). Context sentences were controlled for the number of syllables and words. The second event was the presentation of the stem of a second sentence (STEM; e.g., “She had brought some…. ”). The stem was displayed in four parts consisting of one or two whole words, each displayed for 250 ms in the center of the screen to avoid eye movements. The stem did not contain the last word of the sentence and it is inferred that the participant would produce a semantic prediction (e.g., “sunscreen”) in a highly predictive item. Therefore, prediction and predictability are measured at the time of the STEM event before the final word. The third event in the trial was the presentation of the final word of the sentence (OUTCOME), which was either likely (50% of trials) or unlikely (50% of trials) given the context. Participants then made a response on an MR-compatible response box to indicate likelihood. Importantly, whether the outcome was likely or unlikely was independent of how predictable the item was. Highly predictable and unpredictable items could be paired with a likely or unlikely outcome. The STEM, and the inferred prediction at its end, is the event of interest in this task. Items were constructed in pairs and triplets so that a similar sentence stem was used for different levels of predictability. The length and linguistic properties of the STEM were therefore well controlled between conditions. Presentation of the outcome and the button press response were modeled as a single event (OUTCOME, 1 s), ensuring that prediction error, motor preparation, and motor activity could not contribute to the hemodynamic response at the time of the STEM. Trials with erroneous responses were excluded from the fMRI analysis. Uniformly distributed variable delays were introduced between context and stem (4.5–10.5 s), between stem and outcome (3–7.5 s), and between outcome and the context event of the following trial (4–10 s). This manipulation ensured that BOLD responses to one event were not contaminated with BOLD response to the previous stimulus (for another example of this technique, see Ramnani and Miall, 2004).

#### Localizer tasks for semantic, phonological, and orthographic working memory

When reading a sentence (or a sentence stem), processes in addition to semantic prediction take place. When reading words, one processes the meaning of these words (attention to semantics). When reading words or pronounceable nonwords, one processes phonological features of these words (attention to phonology). When looking at words or nonwords, one recognizes and processes a visual stimulus with a certain spatial configuration (attention to orthography or visuospatial attention). To assess whether any cerebellar areas that respond differentially to predictive sentences were also engaged preferentially when semantic, phonological, or orthographic properties were held in short-term store, participants also performed three epoch-related localizing tasks.

To maximize comparability between tasks and to have a low level of working memory load, all three tasks were 1-back tasks and were contrasted with 0-back versions of the same task. The participants were required press a button on an MRI-compatible response box if the current stimulus belonged to the same semantic category as the previous stimulus (semantic 1-back), if the current stimulus rhymed with the previous stimulus (phonological 1-back) or if the current stimulus was identical to the previous stimulus (orthographic 1-back). Similar tasks have previously been used to capture orthographic and phonological processing (Paulesu et al., 1993; Koyama et al., 2013). Three 0-back control conditions required the participants to respond when a known target stimulus appeared. The 0-back control blocks were performed as separate runs from the 1-back blocks.

##### Semantic 1-back.

**Figure 2.**
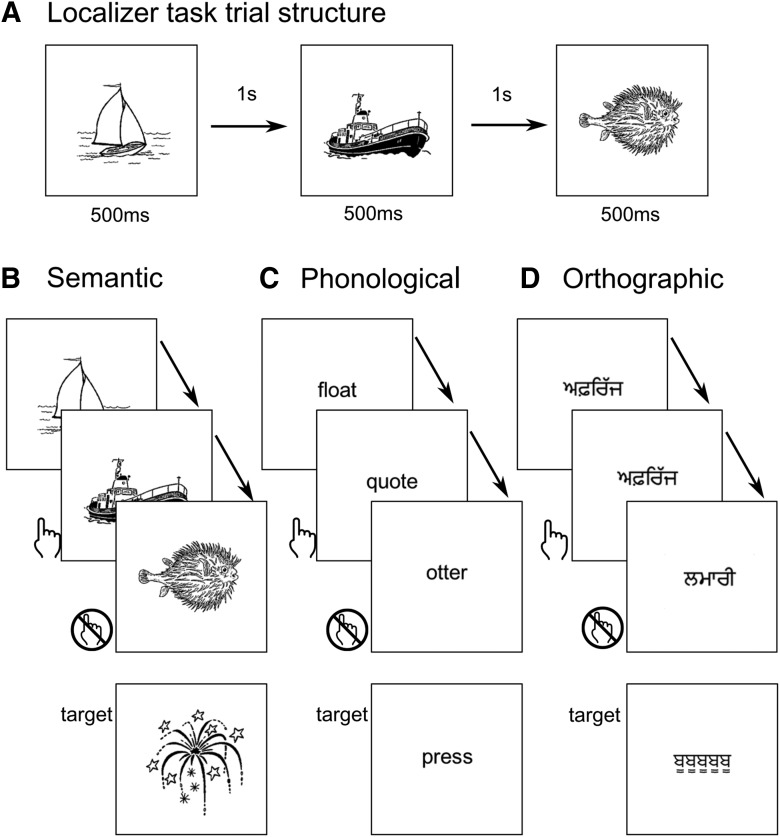
Trial structure and typical stimuli for the localizer tasks. ***A***, Stimulus timing. ***B***–***D***, Top overlapping panels show typical stimulus displays (stimuli presented 500 ms, 1 s apart), illustrating first a match and then a non-match trial, for the 1-back runs. The separate bottom shows the target item for the 0-back runs.

For the semantic task, stimuli were 50 black-and-white line drawings. Participants were familiarized with the 10 stimulus categories (cycles, birds, boats, dogs, fish, fruits, buildings, shoes, tools, and furniture) and the five members of each category, as well as with the 0-back task target object, before scanning (Fig. 2*A*). In contrasting the 1-back with the 0-back condition, we controlled for visual processing of the line drawings and motor activity related to button presses. The requirements that separated the 1-back condition from the 0-back condition were that, in the 1-back condition, participants had to categorize each stimulus, keep this semantic category in short-term memory, and match it to the semantic category of the subsequent stimulus. In the 0-back condition, it was not necessary to process the meaning or semantic category of the line drawing, merely to match it to a target image. We chose line drawings instead of words to avoid automatic phonological processing; line drawings hold meaning but are nonverbal. Nevertheless, we cannot exclude that participants formed a phonological code of the stimulus or the semantic category.

##### Phonological 1-back.

For the phonological task, stimuli were five-letter words printed in the middle of the screen (Fig. 2*C*). Before the scanning session, participants were shown some example stimuli for this task that were not used in the scanning task. They were also shown the target stimulus for the 0-back task. The task was constructed such that a small minority of the rhyming pairs ended in the same syllable. This task could therefore not be performed to an acceptable standard by using a visual search strategy. The 1-back and 0-back conditions were controlled for reading requirements (each condition required reading 5-letter words), demands on attention to meaning or semantics (both conditions likely automatically elicited semantic processing but neither condition required it), and motor activity related to the button presses. Unlike the 0-back condition, the 1-back condition required participants to update and hold the phonetic form of each stimulus in short-term storage and match it to the phonetic form of the subsequent stimulus. The 0-back condition merely required the subject to hold in memory and respond to one target word (the word “press”) throughout the run.

##### Orthographic 1-back.

For the orthographic (visuospatial) task, stimuli consisted of a set of 10 five-letter Punjabi pseudowords (Fig. 2*C*). These stimuli had a similar configuration as written English words, but held no meaning and were not pronounceable for the participants. Participants were familiarized with all the visual stimuli, as well as with the target stimulus for the 0-back task before scanning. Both conditions were matched for low-level visual demands as well as motor activity related to button presses. As in the phonological task, the difference in requirement for the 1-back condition was the higher short-term memory load to retain the visuospatial configuration of each stimulus and compare it with the subsequent stimulus, whereas the 0-back condition required only one easy-to-identify stimulus to be retained throughout.

Each of the 6 runs (3 tasks, each as 1-back and 0-back) lasted 8 min and contained 15 epochs. Each epoch consisted of 10 stimuli and lasted 15 s. Stimuli were presented for 500 ms and were 1000 ms apart. Rest periods between blocks lasted 13–17 s. These rest periods (53% of the scan) were used as an implicit baseline in the analysis.

#### MRI data acquisition

Each participant underwent two fMRI scanning sessions on separate days. One session consisted of a prediction task, divided into three runs each lasting 10 min 30 s. A high-resolution structural image (T1 weighted image, FTE sequence, voxels 1 × 1 × 1 mm) was also collected during this session. During a second fMRI session, participants performed three localizer tasks designed to probe attention to semantic, phonological, and orthographic features of visually presented stimuli. Localizer tasks were divided into an experimental run (1-back condition) and a control run (0-back condition), with each run lasting 8 min. Runs were presented in the same order for each participant. All images were acquired on a 3 T Philips Achieva scanner using a 32-channel head coil (functional: ascending EPI sequence, TR = 3 s, TE = 32 ms, 52 axial slices (no gap), voxels 3 × 3 × 3 mm, FOV 240 × 240, flip angle = 85°). Pulse oximetry and breathing measures were collected with a Philips integrated physiological monitoring system.

#### Statistical analysis of behavior

Behavioral data were processed using custom-made MATLAB scripts (RRID:SCR_001622). Performance in the outcome phase of prediction task and in the localizer tasks was analyzed in R (RRID:SCR_001905) using the packages afex and phia. For the prediction task, a generalized linear mixed model (random intercept, accuracy as binomial dependent variable) was performed with predictability (continuous cloze probability) and outcome (levels: likely and unlikely) as independent variables. For the localizer tasks, a general linear mixed model (random intercept) was used with factors task (levels: semantic, phonological, and orthographic) and condition (levels: 1-back and 0-back). Significant interactions were followed up by *post hoc* tests. Average performance was assessed in all conditions to ascertain that participants paid attention to the task and to allow the exclusion of erroneous trials from the imaging analysis of the prediction task.

#### Statistical analysis of fMRI data

##### Preprocessing.

All analyses were performed in SPM8 (RRID:SCR_007037). Before the first-level analysis, raw images were realigned to correct for head motion, slice-time corrected, and coregistered to the anatomical image. First-level analyses were performed in subject-specific space. Contrast images from the first-level analysis were normalized to the SPM8 EPI template (whole-brain analysis) and smoothed with an 8 mm FWHM Gaussian smoothing kernel before entering group-level analysis. To facilitate later region-of-interest analyses, all EPI images were also normalized and smoothed. The BOLD signal around the brainstem and cerebellum can be vulnerable to confounding physiological signals, but this can be accounted for by regressing out heart rate and breathing signals in the GLM model (Schlerf et al., 2012). The Physiological Log Extraction for Modeling (PhLEM) toolbox in SPM (Verstynen and Deshpande, 2011) was used to convert heart rate and breathing traces into SPM regressors with the CETROICOR method (Glover et al., 2000), resulting in eight regressors that were included as regressors of no interest. Physiological measures from one participant during the control sessions were not available; this person's data were excluded from the analysis of the control tasks.

##### First-level analysis.

For the linguistic prediction task, six events per block were modeled at the first level: context and context_mod_ (a parametric modulator of the context by cloze probability), stem and stem_mod_ (a parametric modulator of the stem by cloze probability), and outcome_likely_ and outcome_unlikely_. The three blocks were concatenated, thus creating a single first-level analysis per person with 18 events of interest. A 19th regressor modeled all trials in which an erroneous response was made to ensure that differences in performance could not underlie differences in BOLD activation patterns. The six contrasts of interest (the six events averaged over the three blocks) were estimated against the implicit baseline. For the localizer tasks, the task blocks were modeled against the implicit baseline in a single *t*-contrast for each of the six sessions. In all tasks, eight regressors of no interest modeled physiological signals and a further six modeled head movement.

##### Group-level analysis.

Normalized first-level contrast images for the prediction task were entered into a factorial design. First, the contrast *t* = [stem] was estimated to assess which regions were engaged in the processing of written meaningful language regardless of predictability (reading contrast). Second, the predictability contrast, *t* = [stem_mod_], revealed areas where the BOLD signal was modulated according to the predictability of the upcoming sentence ending (predictability contrast). A mask of the subjects' brains was created by averaging the normalized skull-stripped anatomical scans coregistered into a 2 × 2 × 2 mm space (216,611 voxels, 1733 cm^3^). A whole-brain cluster correction at a familywise error rate of 5% for this volume was calculated using the 3dclustsim algorithm (Cox, 1996). This procedure determined a voxel-level correction of *p* < 0.001, with a minimum cluster size of 99 voxels (790 mm^3^). In addition, we assessed whether cortical activations were in regions that are functionally connected with the cerebellar region of interest. To this end, resting-state connectivity maps with right Crus I and right Crus II (Bernard et al., 2012; maps provided by the authors) were summed and smoothed with a 4 mm FWHM Gaussian smoothing kernel (see Fig. 4*C*). This resulting connectivity map was then overlaid with the activation map from the predictability contrast.

#### Region of interest (ROI) analyses on areas engaged in prediction

We conducted ROI analyses to determine whether any cerebellar areas that are engaged in linguistic prediction also show increased activity when this prediction is violated (i.e., when the outcome is unlikely versus when it is likely; a prediction error). Moreover, we further assess whether these cerebellar areas were engaged in semantic, phonological, or orthographic processing in the three localizer tasks. ROI analyses were conducted using the marsbar toolbox in SPM8 (Brett et al., 2002). ROIs included cerebellar clusters that were modulated by predictability (predictability contrast), as well as cerebellar areas that were modulated by the presentation of written language (reading contrast). Given our a priori right cerebellar hypothesis, we planned to Bonferroni correct for the number of right cerebellar clusters that are identified in each contrast. To determine whether the activation patterns identified in the cerebellum were unique to this structure or whether cerebral areas also showed the same patterns, we also plotted these parameter estimates of the supratentorial clusters identified in the prediction contrast. These further ROI extractions are strictly exploratory and their results should not be interpreted. Masks of the areas were created by taking a 10 mm sphere around the peak coordinate. First-level design matrices were accessed by marsbar to extract the contrasts estimates for the ROIs defined by the main analysis. This resulted in one parameter estimate per participant per event per ROI.

##### Prediction error analysis.

If linguistic internal models are present in the posterolateral cerebellum, then one might expect these regions to respond more strongly to the unlikely outcomes (prediction error) than to the likely outcomes, analogous to the high activations seen when movement errors occur in motor tasks (Imamizu et al., 2000; Miall et al., 2001). The first-level design matrix from the main prediction analysis was used to extract parameter estimates for Outcome_unlikely_ and Outcome_likely_ events, which were then compared with a paired *t* test. An unlikely outcome does not mean that no prediction was made; it merely means that the outcome violates expectations. A stronger response to unlikely versus likely outcomes indicates that this region processes prediction errors. We hypothesized that those cerebellar areas that are modulated by predictability also respond more strongly when a prediction is violated. A likely or unlikely outcome was equally probable regardless of the item's predictability. This contrast was therefore independent from the predictability contrast.

##### Localizer tasks: attention to semantics, phonology, and orthography.

Given the recruitment of the posterolateral cerebellum in working memory tasks, we were interested in determing whether those regions that are engaged differentially in linguistic prediction are also active in tasks that require short-term storage of semantic, phonological, or orthographic stimulus features. Such functional overlap can provide us with insight into how the cerebellum contributes to language function and how linguistic and working memory contributions may be reconciled. First-level design matrices were created modeling the six conditions (1-back and 0-back conditions for the 3 localizing tasks) individually against the implicit baseline. Parameter estimates were extracted using marsbar and paired *t* tests assessed whether the ROIs showed a larger response to the 1-back condition than to the 0-back condition in the semantic, phonological, and visual localizer. Data from the localizer tasks resulted from independent datasets (from the same participants). Circularity was therefore not a concern.

## Results

### Behavioral results

**Figure 3.**
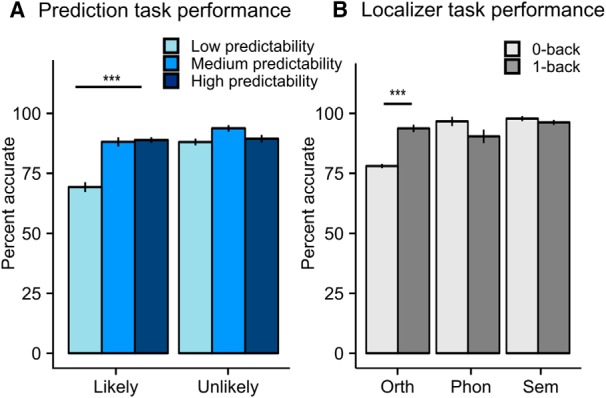
Behavioral performance. ***A***, Percentage accurate responses in prediction task. Discrete levels of predictability were used for display purposes only; analyses were conducted using predictability as a continuous variable. ***B***, Percentage accurate responses in localizer tasks. Error bars indicate ± 1 SEM. ***p < 0.001.

Overall, participants performed well (average 86% correct, SEM = 2.5%, range 79–90%), indicating that all participants were attentive and able to judge whether a sentence ending was likely or unlikely in the context of the trial. The mixed-model ANOVA showed a significant effect for predictability (χ^2^_(1)_ = 17.69, *p* < 0.001), outcome (χ^2^_(1)_ = 15.48, *p* < 0.001), and their interaction (χ^2^_(1)_ = 8.24, *p* = 004). *Post hoc* tests reveal that predictability did not affect performance on unlikely trials (χ^2^_(1)_ = 0.30, *p* = 0.582), but did affect performance on likely trials (χ^2^_(1)_ = 28.69, *p* < 0.001; Fig. 3*A*). These results suggest that a likely sentence ending is less likely to be perceived as such when a prediction is harder to make. Trials with incorrect or missing responses were excluded from the neuroimaging analysis.

On the localizer tasks, participants performed well in all conditions (Fig. 3*B*, average hits 92%, SEM = 2.3%, range 80–96%). The mixed-model ANOVA revealed significant effects of Condition (*F*_(1,80)_ = 4.84, *p* = 0.03), task (*F*_(2,80)_ = 44.76, *p* < 0.001), and the interaction between condition and task (*F*_(2,80)_ = 17.60, *p* < 0.001). Follow-up tests showed that these effects were driven by overall slightly poorer performance in the orthographic task than the other tasks (orthographic vs phonological: χ^2^_(1)_ = 63.17, *p* < 0.001; orthographic vs semantic: χ^2^_(1)_ = 70.88, *p* < 0.001), and poorer performance in the orthographic 0-back task than in the 1-back task (χ^2^_(1)_ = 36.01, *p* < 0.001). No significant differences were present in performance between the phonological and the semantic localizers, or between 1-back and 0-back conditions of these tasks (Fig. 3*B*). These results suggest that the orthographic (visuospatial) localizer was more difficult than the other two tasks.

### Imaging results

#### Areas that respond to written meaningful language (reading contrast)

**Figure 4.**
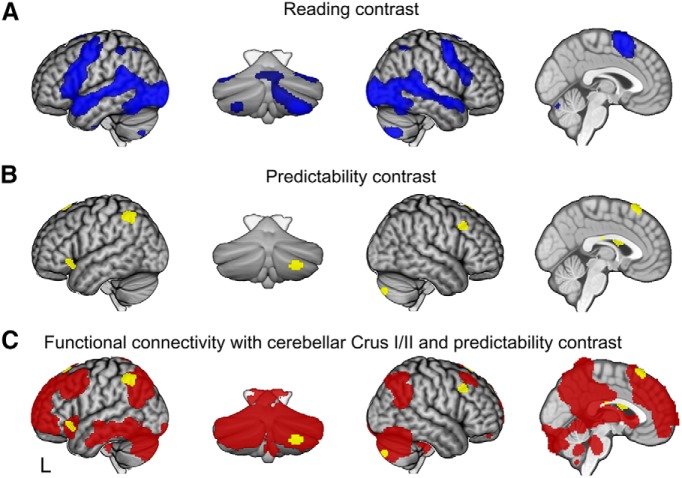
Imaging results. ***A***, Areas activated over baseline when reading (reading contrast). ***B***, Areas where BOLD response is modulated by predictability of future outcome (predictability contrast). Contrasts with familywise error corrected at α < 0.05 (voxelwise *p* < 0.001, cluster size > 99 voxels). ***C***, Areas functionally connected to right Crus I and Crus II based on Bernard et al. (2012) are shown in red, with the results from the predictability contrast (yellow) overlaid to indicate overlap.

The reading contrast revealed a widespread network of cortical and subcortical regions that are classically implicated in language processing, attention, and visual processing (Price, 2012; Rodd et al., 2015). Areas engaged when processing the sentence stem were bilateral inferior and middle frontal gyrus, medial frontal gyrus, bilateral middle temporal gyrus extending from the temporal pole into temporoparietal cortex, left thalamus, bilateral posterolateral cerebellum, and the cerebellar vermis (Fig. 4*A*, Table 1). Activations were more pronounced on the left of the cerebral cortex and on the right in the cerebellum.

**Table 1.** Imaging results

Gross anatomical region	Volume (mm^3^)	T	MNI coordinates			Cytoarchitectonic region
			*x*	*y*	*z*	
**Reading contrast**						
**Frontal**						
Left inferior frontal gyrus	179,384 (5)	10.57	−50	12	24	BA 44
Left precentral gyrus	179,384 (6)	9.87	−50	−6	52	BA 6
Right inferior frontal gyrus	32,440 (1)	6.03	48	18	24	BA 44
Right middle frontal gyrus	32,440 (2)	8.48	50	2	56	BA 6
Left superior frontal gyrus	17,592	13.19	−6	8	56	BA 6/SMA
**Parietal**						
Right inferior parietal lobule	12,608	8.94	32	−52	46	BA 7
Left inferior parietal lobule	12,472	6.21	−34	−58	50	BA 7
**Occipital**						
Left middle occipital gyrus	179,384 (1)	14.69	−36	−94	−4	hOC4v (V4)
Left inferior occipital gyrus	179,384 (3)	11.98	−44	−60	−14	BA 37
Right inferior occipital gyrus	118,064 (1)	12.25	42	−92	−2	hOC3v (V3v)
Right inferior occipital gyrus	118,064 (3)	11.3	28	−94	−4	BA 18
**Temporal**						
Left inferior temporal gyrus	179,384 (2)	13.14	−40	−46	−16	BA 37
Left middle temporal gyrus	179,384 (4)	11.65	−54	−50	12	BA 21
Right inferior temporal gyrus	118,064 (2)	12.56	44	−62	−12	BA 37
Right middle temporal gyrus	118,064 (4)	9.99	54	−36	4	BA 22
Right superior temporal gyrus	118,064 (6)	6.92	60	2	−14	BA 22
Left temporal pole	179,384 (7)	9.33	−52	10	−20	BA 38
Left middle temporal gyrus	179,384 (8)	8.99	−56	−6	−12	BA 22
**Insular regions**						
Right insula lobe	2,552	6.15	34	24	4	
**Cerebellum**						
Right cerebellum	118,064 (5)	9.59	30	−70	−52	Lobule VIIb (Hem)
Right cerebellum	118,064 (7)	6.37	30	−62	−26	Lobule VI (Hem)
Left cerebellum	1,304	5.68	−30	−70	−52	Lobule VIIb (Hem)
**Other subcortical**						
Left thalamus	840	4.55	−8	−16	12	
**Predictability contrast**						
**Frontal**						
Left superior frontal gyrus	1,584	4.43	0	28	62	BA8/pre-SMA
Left inferior frontal gyrus	1,592	4.14	−42	22	−10	BA47
Right middle frontal gyrus	1,360	4.66	44	20	40	BA 9/46
**Parietal**						
Left superior parietal lobule	1,600	4.04	−50	−58	56	BA 7
**Cerebellum**						
Right cerebellum	1,072	4.19	28	−86	−48	Lobule VIIa Crus II (Hem)
**Other subcortical**						
Right caudate nucleus	2,664	4.76	6	4	18	

Data are cluster corrected (FWE-corrected alpha < 0.05: voxelwise *p* < 0.001, cluster size > 99 voxels). For clusters that encompass multiple peaks, the volume of the entire cluster is given, with the index of the subpeak in parentheses.

#### Areas where activity covaries with predictability (predictability contract)

**Figure 5.**
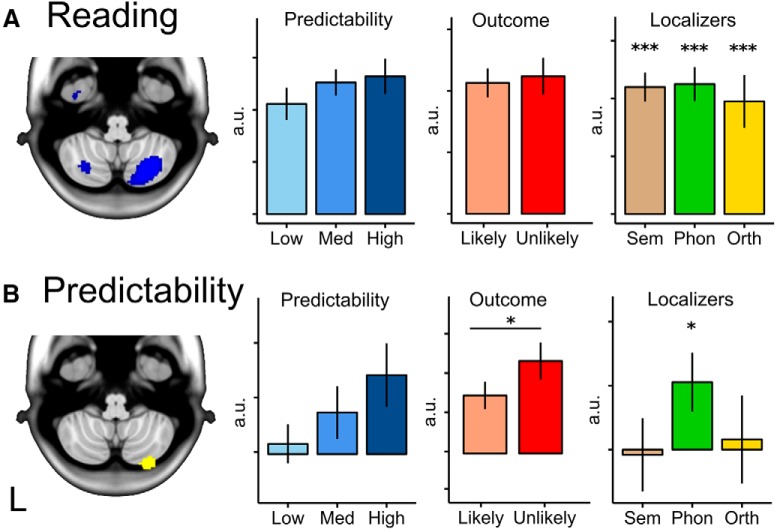
Parameter estimates for right cerebellar activations. First column: Cerebellar clusters in reading contrast (***A***, blue) and predictability contrast (***B***, yellow) whole-brain corrected at FWE *p* < 0.05. Second column: Predictability. Parameter estimates were extracted for different levels of predictability. Regression weights were extracted from the clusters identified in the imaging analysis where predictability (cloze probability) was a continuous variable. These are plotted to aid interpretation only; no statistical inference should be drawn. Third column: Outcome. ROI analysis for prediction error using clusters as ROIs is shown. Fourth column: Localizer task responses. Shown is ROI analysis for semantic, phonological, and orthographic processing (1-back − 0-back) using the same cluster masks. *p < 0.05, ***p < 0.001, paired *t* tests. a.u., Arbitrary units. Error bars indicate SEM.

**Figure 6.**
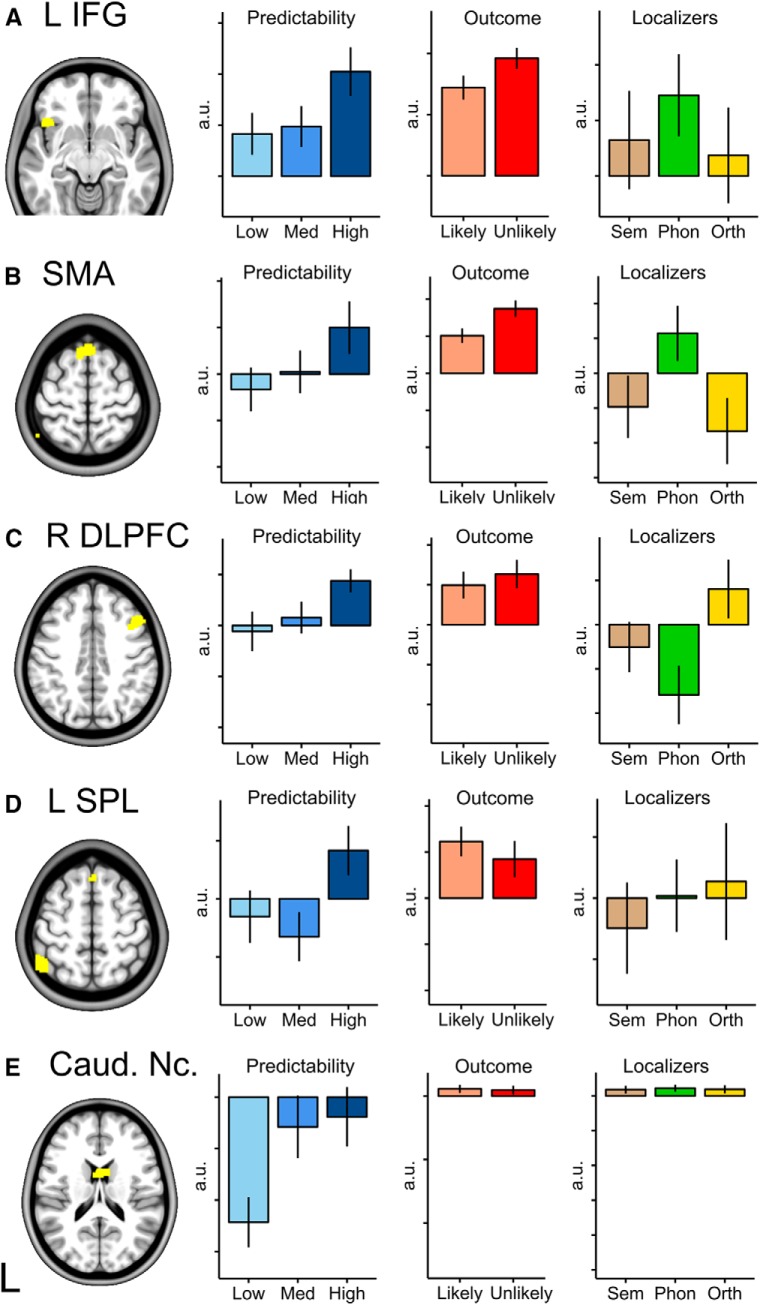
Parameter estimates for cerebral areas engaged in prediction. Left, Supratentorial brain areas that relate to predictability whole-brain corrected at familywise error *p* < 0.05. Columns 2–4: Predictability. Parameter estimates were extracted for varying levels of predictability (see Fig. 5 for details). Regression weights were extracted from the clusters identified in the imaging analysis where predictability (cloze probability) was a continuous variable. These are plotted to aid interpretation only; no statistical inference should be drawn. Outcome: parameter estimates for prediction errors; because these areas were not part of an a priori hypothesis, no statistical inference should be drawn. Localizers: parameter estimates (1-back minus 0-back) for semantic, phonological, and orthographic processing; again, because these areas were not part of an a priori hypothesis, no statistical inference should be drawn. a.u., Arbitrary units. Error bars indicate SEM.

The predictability contrast revealed an area in right posterolateral cerebellum, Crus II, where hemodynamic activity positively correlated with predictability (Figs. 4*B*, 5*B*, Table 1). Supratentorial clusters were identified in the left inferior frontal gyrus, right middle frontal gyrus, left posterior parietal cortex, presupplementary motor area, and right caudate nucleus (Figs. 4*B*, 6, Table 1). No brain areas showed activity that correlated negatively with the predictability of the items. All clusters apart from the right middle frontal gyrus cluster overlapped with a map of regions that are functionally connected to Crus I and Crus II (Fig. 4*C*).

#### ROI analyses: cerebellar area that represent prediction also represent prediction error

A paired-samples *t* test compared the regression weights for unlikely outcomes and likely outcomes for the cerebellar cluster that was modulated by predictability (predictability contrast) and for the cerebellar area that responded to written language (reading contrast). Because only one cluster was identified in each contrast, tests were considered significant at *p* < 0.05. The Crus II cluster that was modulated by predictability (Fig. 5*B*) also showed a larger response to unlikely than to likely sentence outcomes (MNI 28,−86,−48 likely > unlikely: *t*_(16)_ = 2.27, *p* = 0.037). Conversely, the larger area that responded to the stem event (Fig. 5*A*) did not show such a difference (MNI 30,−70,−52: *t*_(16)_ = 0.33, *p* = 0.743).

#### ROI analyses: cerebellar area that represents predictability is engaged in phonological processing but not semantic or orthographical processing

Paired *t* tests compared the activity in 0-back and 1-back conditions for semantic, visual, and phonological localizers. This analysis indicates whether the areas that were modulated by predictability were also engaged by attention to semantic content, phonological, or visual features. Results showed that right Crus II was significantly engaged in the phonological localizer task (MNI 28, −86, −46; *t*_(15)_ = 2.52, *p* = 0.032), but not in the semantic or orthographic task (Fig. 5*B*). Note that these results do not imply that the Crus II region is more engaged in the phonological task as compared with the other two tasks. The condition effect (1-back minus 0-back) in the phonological task differs from that in the semantic task (*t*_(15)_ = 2.49, *p* = 0.025), but not from that in the orthographic task (*t*_(15)_ = 1.23, *p* = 0.238). However, these between-task comparisons do not survive a Bonferroni correction for the three possible post-hoc tests. The Crus II region that responded to written language (reading contrast) was significantly recruited in all three localizer tasks (MNI 30, −70, −52; semantic: *t*_(15)_ = 8.82, *p* < 0.001; phonological: *t*_(15)_ = 9.08, *p* < 0.001; orthographic: *t*_(15)_ = 7.43, *p* < 0.001). This activation pattern is consistent with a region that is engaged in processing written meaningful language because this entails semantic, phonological, and orthographic processing.

In summary, we found that a discrete region in cerebellar Crus II was significantly modulated by the predictability of the stem sentence in the interval before the outcome was presented. This area was also active in a contrast that probed phonological processing, but not in contrasts that probed semantic or visual processing. It lay within a broader zone of the cerebellum activated by the reading task (but not modulated by predictability) and that broader zone did overlap with the regions activated by semantic and orthographic processing.

## Discussion

The right posterior cerebellum is consistently implicated in language processing, but its precise contribution remains unclear. In parallel with the predictive function of cerebellar motor regions through internal models of movements (Courchesne and Allen, 1997; Miall, 1998; Ebner and Pasalar, 2008), internal model prediction may generalize to nonmotor cerebellar regions, particularly Crus I/II (Ramnani, 2006; Ito, 2008). Therefore, language-sensitive right cerebellar regions may assist linguistic processing by predicting upcoming sentence content. Here, we tested this hypothesis with a closely controlled event-related fMRI study. We compared activity time locked to the presentation of identical sentence fragments that varied in the degree to which they predicted the final word of the sentence (their cloze probability). Crucially, this sentence fragment was modeled independently from a context sentence through which predictability was manipulated and from the final word (outcome) of the sentence. We were thus able to capture effects of prediction in the absence of outcome evaluation or prediction error while also avoiding motor, linguistic, and working memory confounds. Further, using separate fMRI localizer tasks, we assessed whether identified prediction-sensitive areas were also engaged in semantic, phonological, or orthographic processing.

As hypothesized, activity in right Crus II increased with the predictability of the upcoming sentence ending. Further consistent with the presence of internal model predictions, the same Crus II area was more active during an unexpected outcome (prediction error) than an expected outcome. Finally, this area was also engaged when attending to phonological information, but not semantic or orthographic information.

This study is the first to identify a right cerebellar region that represents predictability independently from motor demands or error processing. Our findings complement and extend existing evidence on linguistic prediction in the right posterolateral cerebellum. Previous fMRI evidence indicates that right posterior cerebellar regions are engaged when a linguistic prediction is possible (Desmond et al., 1998; Moberget et al., 2014). We have shown previously that low-frequency right cerebellar rTMS disrupts the prediction of upcoming sentence content in a language comprehension task (Lesage et al., 2012), a finding that we replicated recently using cathodal tDCS (Miall et al., 2016). In the language production domain, rTMS over right, but not left, cerebellum impairs higher level speech monitoring, including internal prediction of upcoming speech (Runnqvist et al., 2016), and a recent study found that right cerebellar tDCS improved performance in a sentence completion task (D'Mello et al., 2017). Such neurostimulation evidence dovetails nicely with the present data to show that the right posterior cerebellum is causally involved in linguistic prediction to aid both language comprehension and language production.

A posterolateral cerebellar contribution to language processing is consistent with the region's connectivity fingerprint. Viral tracer studies in nonhuman primates (Middleton and Strick, 1998; Kelly and Strick, 2003; Akkal et al., 2007; Bostan et al., 2013) and resting-state functional connectivity and meta-analytic connectivity mapping in humans (Habas et al., 2009; Krienen and Buckner, 2009; Buckner et al., 2011; Bernard et al., 2012; Balsters et al., 2014) have identified connectivity between Crus I/II and higher-order cognitive and language regions, including inferior frontal, dorsolateral prefrontal, posterior parietal, and anterior cingulate cortices. In the present data, cerebral areas where the hemodynamic response scaled with linguistic predictability included the left inferior frontal gyrus, pre-SMA, left posterior parietal lobe, right middle frontal gyrus, and bilateral caudate nucleus. These areas are all implicated in lexicosemantic or phonological language processing (Fedorenko et al., 2010; Wu et al., 2012; Martin et al., 2015) and all except the right DLPFC cluster were within the network of regions functionally connected to right Crus I/II (Bernard et al., 2012).

Our findings support the idea that cerebellar internal models aid language comprehension by predicting upcoming stimuli. Internal models are prominent in theories of motor cerebellar function (Miall, 1998; Wolpert et al., 1998) and it has long been hypothesized that cognitive and linguistic internal models could be present in prefrontal-projecting cerebellar areas (Leiner et al., 1989; Ramnani, 2006; Ito, 2008). Internal model prediction has been incorporated into psycholinguistic accounts more recently (Hickok, 2012; Rothermich and Kotz, 2013; Kotz et al., 2014; Pickering and Garrod, 2014). One fairly comprehensive theoretical framework posits that comprehension is achieved using the speech production apparatus, with both speech production and comprehension aided by internal model prediction (Pickering and Garrod, 2013; Pickering and Clark, 2014). This model aligns well with our present findings and previous neurostimulation and neuroimaging evidence (Lesage et al., 2012; Moberget et al., 2014; Miall et al., 2016; D'Mello et al., 2017), which indicate that prediction of upcoming words may occur in or depend upon the cerebellum.

A major challenge in determining the function of prefrontal-projecting cerebellar areas is their involvement in processes that are difficult to manipulate separately. Notably, the Crus I/II area implicated in language is also consistently implicated in verbal working memory, where recruitment scales with cognitive load (Hayter et al., 2007; Lesage et al., 2010; Marvel and Desmond, 2010, 2012). Indeed, it has been proposed that the posterior cerebellum may act as Baddeley and Hitch's (1974) phonological store, encoding verbal content and keeping this information online (Chen and Desmond, 2005; Marvel and Desmond, 2010). However, the involvement of the posterior cerebellum in language cannot be explained entirely by working memory demands. The right posterolateral cerebellum is recruited consistently in lexicosemantic processing (Vandenberghe et al., 1996; Fedorenko et al., 2010; Price, 2012; Lesage et al., 2016), even in relatively undemanding conditions such as reading meaningful sentences, in contrast to more cognitively demanding scrambled sentences (Moberget et al., 2014). To explore functional overlap between working memory and language processes, we assessed cerebellar recruitment in 3 1-back tasks that each captured a component of reading; attention to semantics (semantic categorization), attention to phonology (rhyming judgment), or attention to orthographic features (visuospatial matching). In the present data, we found that the prediction-sensitive cerebellar cluster was engaged in the phonological task, but we did not find that this area was engaged in the semantic or orthographic tasks. This area's recruitment in a phonological task aligns with a cerebellar role in the phonological store and inner speech (Ackermann et al., 2004, 2007, Marvel and Desmond, 2010, 2012). The absence of this area's significant engagement in the semantic task is somewhat surprising, especially because evidence for cerebellar linguistic prediction is largely derived from semantic prediction tasks, including the task used here (Lesage et al., 2012; Argyropoulos, 2016; Miall et al., 2016; D'Mello et al., 2017). However, our data do not necessarily mean that internal models exclusively predict the phonological form of upcoming content or that this prediction cannot be semantic. For example, semantic predictions may be represented in a common code to the representations needed in the phonological task. Alternatively, the semantic task, which used line drawings, may have captured semantic processes distinct from those in the prediction task and a different localizer task might have recruited the prediction-sensitive cerebellar region.

A larger area of right Crus II that was activated consistently during reading (when meaningful language was presented) but not specific to prediction, was engaged robustly in all three localizer tasks. This is consistent with imaging evidence for semantic processing in posterolateral cerebellum (Price, 2012) and with meta-analyses of cerebellar recruitment in various tasks, where clusters responding to verbal working memory and language tasks overlap (Stoodley and Schmahmann, 2009, 2010; Keren-Happuch et al., 2012; Stoodley et al., 2012).

This study is not without limitations. First, stimulus type differed between the localizer tasks. Even though the contrasts used controlled for such lower-level differences, it is possible that a semantic localizer using written language might have produced different results, potentially recruiting the cerebellar area that scaled with predictability. Second, the analysis on the localizer tasks is unable to speak to whether regions are recruited differently in different localizer tasks. Third, the order of the localizer task runs was not counterbalanced. We can therefore not exclude fatigue or learning effects. However, given the lack of performance differences, we think it unlikely that order affected the phonological or semantic localizer tasks. Lower performance in the 0-back condition of the orthographic localizer may be partially attributable to fatigue, but it is not clear what outcome such an order effect would have on cluster location.

Future research can further elucidate how working memory and linguistic prediction are represented in the cerebellum and whether internal model prediction could be an underlying mechanism to support these functions. Tasks using different stimulus types may shed further light on how linguistic prediction takes place in the cerebellum. Finally, study of the interaction between supratentorial areas that are functionally connected to the cerebellum and also represent predictability can elucidate how linguistic internal model prediction is achieved.

### Conclusions

We identified an area in cerebellar Crus I/II where BOLD response scales with the predictability of upcoming sentence content. Activity in this region was larger when an unexpected sentence ending was evaluated compared with an expected sentence ending, consistent with processing prediction errors. Interestingly, the cerebellar area modulated by predictability was also recruited in a phonological processing task, but not in orthographic or semantic processing tasks. Therefore, our results support the presence of linguistic internal models during language comprehension and suggest that this process may rely on phonological processing.
